# A rapid-release pure iodine coating on titanium implants to mitigate acute periprosthetic infections

**DOI:** 10.3389/fbioe.2025.1590411

**Published:** 2025-07-10

**Authors:** Yong-Quan Zhang, Jie Liang, Qing-Quan Chen, Xiao-Li Huang, Wan-Ming Wang, Jin-Shui Chen, Xiu Yang

**Affiliations:** ^1^ Fuzong Clinical Medical College of Fujian Medical University, Fuzhou, China; ^2^ The School of Basic Medical Sciences, Fujian Medical University, Fuzhou, China; ^3^ Institute of Laboratory Animal Center, Fujian Medical University, Fuzhou, China

**Keywords:** iodine, titanium, orthopedic implants, infections, biofilm prevention

## Abstract

**Background:**

Periprosthetic infections remain a significant challenge in orthopedic surgeries, primarily due to bacterial biofilm formation on implant surfaces. To address this issue, we developed a novel iodine-based coating on titanium implants designed to rapidly release iodine, thereby preventing acute infections. The efficacy and safety of this coating were assessed through both *in vitro* experiments and an *in vivo* rabbit model.

**Methods:**

The iodine coating was applied to titanium implants using electrophoretic deposition. The coated implants were characterized using scanning electron microscopy (SEM), X-ray fluorescence spectroscopy (XRF), and energy-dispersive spectroscopy (EDS). *In vitro* studies included antibacterial assays, iodine release kinetics, and hemolysis tests. Additionally, an acute periprosthetic infection model in rabbits was established to evaluate the coating’s performance *in vivo*.

**Results:**

The electrophoretic deposition technique successfully produced a uniform iodine coating with high iodine content and rapid release kinetics. *In vitro* tests demonstrated significant antibacterial activity against *Staphylococcus aureus* and *Escherichia coli*. The rabbit model showed a marked reduction in infection rates compared to uncoated implants, with no adverse effects on bone integration.

**Conclusion:**

This study introduces a promising iodine-based coating for titanium implants, offering a rapid and effective solution to prevent acute periprosthetic infections while maintaining biocompatibility and supporting bone healing.

## 1 Introduction

Implant-related infection is a common and devastating complication of various orthopedic surgeries. In the aging societies of many countries worldwide, arthroplasty and orthopedic internal fixation procedures are being increasingly performed, among which periprosthetic joint infections contribute to failure rates exceeding 1% for hip arthroplasty and ranging from 1% to 3% for knee arthroplasty (Kurtz et al., 2010; Kapadia et al., 2016; Dale et al., 2021). Furthermore, the incidence of postoperative infection following spinal surgery ranges from 2% to 5% (Zhou et al., 2020), while that after limb fractures varies from 5% to 15% (Depypere et al., 2020). The bacteria responsible for plant infections in orthopedics have been identified in clinical studies, with coagulase-negative *Staphylococcus* (30%–43%), *Staphylococcus aureus* (12%–23%), *Streptococcus* (9%–10%), *Enterococcus* (3%–7%), Gram-negative bacteria (3%–6%), and anaerobic bacteria (2%–4%) being the most prevalent pathogens (Akkaya et al., 2023). Implant-associated infection leads to increased medical costs, disability, and fatality rates; affects the quality of life of patients; and causes a heavy social burden.

The formation of a bacterial biofilm on the surface of invasive orthopaedic-surgical implants and bacterial colonization in the bone tissue constitute the primary etiological factors underlying implant-related infection in orthopedics (Izakovicova, Borens, and Trampuz, 2019; Yang et al., 2018; Jensen et al., 2017). The adhesion and colonization of bacteria in host tissues are critical steps in the pathogenesis of plant-associated infections in orthopedics. Current therapeutic outcomes of plant infection treatment in orthopedics, which typically involve surgical debridement, implant removal, and long-term antimicrobial therapy, are suboptimal from a clinical perspective (Nelson et al., 2023). As such, prioritization of the inhibition of the initial bacterial adhesion to internal plant surfaces has emerged as a paramount concern.

Modifications of the antibacterial coating on the inner surface of implants can effectively eliminate bacteria or hinder their adhesion upon contact with the inner plant. In accordance with the mechanism of action exhibited by antibacterial coatings, they can be broadly categorized into three main types (Chouirfa et al., 2019): 1) Passive surface modification: By altering the surface characteristics of the implant, such as roughness, hydrophilicity, surface energy, and conductivity, it is possible to prevent or reduce bacterial adhesion without releasing bactericidal substances into the surrounding tissue. For example, the application of polyethylene glycol to a titanium alloy surface significantly inhibits bacterial adhesion. 2) Active surface modification: Incorporating pharmacologically active antibacterial agents, such as metals (e.g., silver, zinc, and copper), non-metallic elements (e.g., iodine and selenium), and organic compounds (antibiotics, anti-infective peptides, chitosan), can effectively eradicate bacteria or inhibit adhesion. Recent advances in surface modification strategies for orthopedic implants emphasize the importance of balancing antibacterial efficacy and biocompatibility. For instance, Geng et al. (2021) developed a novel snail-inspired bionic design of titanium with strontium-substituted hydroxyapatite coating for promoting osseointegration (Geng et al., 2021). Similarly, bioactive coatings such as selenium-doped hydroxyapatite have shown dual functionality in inhibiting bacterial adhesion and promoting bone regeneration (Geng et al., 2022). Sang et al. reported a Sponge-inspired sulfonated polyetheretherketone loaded with polydopamine-protected osthole nanoparticles and berberine enhances osteogenic activity and prevents implant-related infections (Sang et al., 2022). 3) Local antibacterial carrier: A biodegradable or non-biodegradable antibacterial carrier, such as polymethyl methacrylate, can be applied to facilitate the adhesion of antimicrobial agents around the implant, thereby achieving an anti-bacterial effect. However, the progress of implant surface coatings *in vitro* and in animal experiments has been limited, with varying degrees of deficiency. For example, although silver ions exhibit excellent antibacterial properties, their cytotoxicity cannot be overlooked, and controlling the release of implant antibiotic coatings remains challenging (Xu et al., 2020; Liu et al., 2021). Currently, antibacterial implant coating materials that are extensively used in practical and clinical settings are lacking.

Povidone-iodine is a widely used surgical disinfectant owing to its high efficacy, rapid antibacterial activity, and low tissue toxicity (Barreto et al., 2020). Iodine, the primary antibacterial component of povidone-iodine, serves as an optimal non-metallic coating material. Currently, both anodic oxidation and electrophoretic deposition methods are employed for iodine doping in titanium dioxide nanotubes, with corresponding fundamental research experiments validating the antibacterial and anti-adhesion properties of the resulting iodine coating (Shirai et al., 2014; Yang et al., 2023). However, the iodine-doped titania nanotube coating still exhibits certain limitations, such as inadequate antibacterial efficacy attributed to its low iodine carrying capacity. Our previous studies have shown that the presence of nanotubes seems to hinder iodine release to some extent, resulting in a prolonged iodine release cycle, with the coating still containing close to 30% iodine after 1 year, which may affect the efficiency of bone integration and reduce the effectiveness of preventing acute periprosthetic infections (Yang et al., 2023). Therefore, for the prevention of acute periprosthesis infection, we need a coating with high iodine content and a relatively short iodine-release cycle, which can effectively prevent infection in the acute phase without affecting bone integration in the later stage. The bone histopathological diagnostic criteria for acute osteomyelitis in this study primarily refer to Sybenga et al. (2020), with the Jupiter scoring system proposed as a pathological tool for diagnosing bone infections (Sybenga et al., 2020). We postulated whether iodine can be directly immobilized on the surface of titanium implants without the necessity to induce nanotube formation on titanium plates. Subsequently, a titanium implant with an iodine coating was developed and its antibacterial performance was verified through *in vivo* and *in vitro* tests. This study aimed to provide a novel approach for the research and development of antibacterial implant in orthopedics.

## 2 Materials and methods

### 2.1 Preparation of iodine-coated orthopedic titanium needle

The experimental orthopedic titanium needle (3.0 cm in length, 2.0 mm in diameter, Ti-6Al-4V alloy, Dabo Medical Technology Co., Ltd., Xiamen, China) was meticulously polished to eliminate any visible scratches. To prepare the coating, 400 mg of solid povidone-iodine powder (Shanghai De Xiang Pharmaceutical Co., Ltd., Shanghai, China) were added to 100 mL of distilled water and thoroughly mixed. A titanium needle served as the anode, and a platinum sheet (4 cm long, 2 cm wide) acted as the cathode. Applying a direct current power supply at 200 V (WYK-6005 K DC power supply, DCSOON, Shenzhen, China) for 30 min drove the formation of iodine-coated orthopedic titanium needles. Subsequently, the coated needles were removed and ultrasonically cleaned with distilled water for 10 min, followed by drying. Coating characterization involved observation under a scanning electron microscope as well as analysis using X-ray fluorescence spectrometry (Philips, Eindhoven, Netherlands) and an energy dispersive spectrometer (EDS) (Philips). The content and distribution of the elements on the coating surface were determined.

### 2.2 *In vitro* antibacterial experiment

The control group (TI) comprised 10 titanium needles without iodine, whereas the experimental group (TI-I) comprised 10 iodine-coated titanium needles fumigated with low-temperature ethylene oxide prior to use. The *S. aureus* standard strain (ATCC25923) was selected, and two bacterial solutions with concentrations of 1*10^6^ colony-forming units (CFUs)/ml were separately prepared. The titanium needles were placed in sterile culture cups and completely immersed in 30 mL of the prepared bacterial suspension, followed by incubation at 37°C for 6 h. After removal, the titanium needles were rinsed with 5 mL phosphate buffered saline (PBS) and transferred to new sterile culture cups. Subsequently, they were fully immersed in 5 mL sterile normal saline and subjected to ultrasonic shaking. The shaken bacterial solution was then diluted by a factor of 10,000 and applied onto a plate counting medium with a diameter of 9 cm for cultivation at 37°C. After incubation for 24 h, the number of CFUs in each plate were counted. A similar method was used to conduct an *in vitro* antibacterial test using an *Escherichia coli* standard strain (ATCC25922).

### 2.3 Iodine release kinetics in an *in vitro* experimental setup

The titanium needle coated with iodine was stored in normal saline at a temperature of 37°C, and the changes in surface iodine were analyzed using X-ray fluorescence spectroscopy (XRF) and an EDS on the 3rd, 7th, and 14th days.

### 2.4 Toxicity test

Titanium needles of equal length, both uncoated and iodine-coated, were immersed in 1 mL of a suspension of 4% rabbit red blood cells (QS004; BioLab, Shanghai, China). Normal saline served as the negative control, and 1% TritonX-100 was designated as the positive control. Following incubation at 37°C for 30 min, a supernatant volume of 100 µL was transferred to a 96-well plate, and the absorbance at 540 nm was measured using a Multiskan SkyHigh microplate spectrophotometer (Thermo Fisher Scientific, Waltham, MA, United States). The hemolysis rate was calculated as follows: hemolysis rate % = (sample - negative control)/(positive control - negative control) *100%.

### 2.5 Reconstruction of a rabbit model of acute periprosthetic bone infection caused by *Staphylococcus aureus* and assessment of antibacterial efficacy *in vivo*

#### 2.5.1 Animal model

Ten iodine-free titanium needles and 10 iodine-coated titanium needles (8 mm in length and 1 mm in diameter) were prepared following the aforementioned method. Subsequently, all titanium needles were subjected to ethylene oxide fumigation. Twenty New Zealand rabbits (male and female) weighing 2.5 ± 0.2 kg were individually housed in cages and acclimated for a period of 7 days prior to the surgical procedure. The rabbits were randomly assigned numbers from 1 to 20 using computer-generated randomization, after which they were divided into two groups: a control group (TI) and an experimental group (TI-I) comprising 10 rabbits each. General anesthesia was induced by administering 30 mg/kg 1% pentobarbital sodium through the auricular vein. Once anesthetized, a rubber band was applied to the base of the left thigh as a tourniquet, followed by routine disinfection of the left lower limb using a solution containing 0.5% iodobenzene. A medial skin incision, measuring approximately 2 cm, was made overlying the metaphyseal region of the left tibia, after which tissue separation was performed. The tibia was accessed using a 2.5-mm electric drill, creating a window with a depth of 10 mm from the inner to outer surface. Subsequently, the titanium needle (8 mm long and 1 mm diameter) was inserted into the opening, followed by inoculation of *Staphylococcus aureus* bacterial solution at a concentration of 1.0 × 10^6^ CFU/mL. Iodine-coated titanium needles were implanted in the experimental group, whereas ordinary titanium needles were used in the control group. After bacterial inoculation, the opening was sealed with bone wax, and the incision was closed using a 3–0 silk thread. The detailed surgical procedure is illustrated in Supplementary Figure S1. Following the surgical procedure, the animals were housed in cages and provided with regular food and water. The wounds were examined daily and treated with appropriate dressings. Gait and feeding conditions were assessed. No postoperative antibiotics were administered to any of the animals, and the observation period lasted for 1 week or until death occurred. The overall condition of the animals was evaluated for signs indicative of infection, including wound swelling, redness, soft tissue damage, and pus discharge. The animals used in this study were obtained from the Animal Center of the 900th Hospital Joint Logistic Support Force. All animal experiments were performed in accordance with the International Association of Veterinary Editors (AVMA) guidelines. The animal study was reviewed and approved by the Experimental Animal Welfare Ethics Committee of the 900th Hospital of the Joint Logistic Support Force (Ethics No. 2020–053).

#### 2.5.2 Microbiological assessment

The experimental rabbits were euthanized via intravenous injection of pentobarbital sodium (100 mg/kg). Subsequently, the secretions surrounding the needle path were inoculated into a standard agar nutrient medium and incubated at 37°C for 24 h to facilitate observation. The bacterial culture results were examined using an automated microbiological identification instrument.

#### 2.5.3 Histopathological evaluation

The affected limb of each rabbit was subjected to severance testing of the knee and ankle joints, followed by cleaning of the surrounding soft tissues and muscles. The intact tibia was then removed, split longitudinally with a bone knife, and examined using scanning electron microscopy and laser confocal microscopy after the removal of the titanium needle. The resulting sections were cut into equal lengths for further examination. A 1-mm sample of spongy bone around the needle canal was fixed in 10% formalin solution for 72 h, decalcified with a 5% formic acid solution, stained with hematoxylin-eosin, and observed under a microscope to detect any infection. Histopathological findings of the bones were assessed using the Jupiter scoring system, which incorporated 15 histological features. A score of ≥6 points, along with the presence of at least one histological feature indicative of acute osteomyelitis, was considered consistent with histopathological evidence supporting a diagnosis of acute osteomyelitis (Sybenga et al., 2020). Histopathological findings were quantitatively assessed by counting neutrophils and monocytes in five randomly selected high-magnification fields (400×) per sample. Neutrophil aggregation was defined as >10 neutrophils per field, based on Sybenga et al. (2020). All counts were performed independently by two blinded pathologists using ImageJ software (NIH, Bethesda, MD, United States), and averages with standard deviations (SD) were calculated for statistical comparison.

#### 2.5.4 Observation by scanning electron microscope

Following 48-h incubation in 2.5% glutaraldehyde solution, the titanium needle was rinsed three times with 0.1 mol/L PBS buffer. Subsequently, fixation was performed for 2 h using 2.5% glutaraldehyde, and dehydration was performed once each with 30%, 50%, 70%, and 80%, and finally, three times with anhydrous ethanol for 10 min each. The surface bacteria on the titanium needles were observed using a scanning electron microscope (Gemini; ZEISS, Oberkochen, Germany) after drying and gold spraying.

#### 2.5.5 Laser confocal microscope observation

The titanium needle was preserved by immersion in a 2.5% glutaraldehyde solution. Subsequently, the Kirschner needle was rinsed three times with PBS. Next, the titanium needle was placed in a dedicated Petri dish for laser confocal microscopy and 1 mL of 2.5% glutaraldehyde was added for fixation at 4°C for 1.5 h, followed by another rinse with PBS buffer. Subsequently, an appropriate amount of prepared green fluorescent nucleic acid dye (SYTO™ 9, Thermo Fisher Scientific, Waltham, MA, United States) was added, and the Petri dishes were incubated at 4°C away from light for 30 min. Finally, two rinses were performed with PBS to eliminate excess SYTO™ 9 dye. Subsequently, an appropriate quantity of propyl iodide dye (Thermo Fisher Scientific) was added and incubated in a light-free refrigerator at 4°C for 15 min. Following two rinses with PBS, the samples were air-dried in the dark at room temperature and subjected to anti-fluorescence attenuation sealing. Finally, the staining was observed using a laser confocal microscope (LSM 980, ZEISS), and detailed image analysis was performed using FV10-ASW 2.0 Viewer software (Olympus, Beijing, China).

#### 2.5.6 Infection rate evaluation

The infection rate of the rabbits in each group was assessed 1 week post-surgery. To diagnose an infection, at least one positive result was obtained using microbiological, histopathological, or other evaluation methods.

#### 2.5.7 Observation of the metaphyseal bone by transmission electron microscopy

A 1 × 1 × 1 mm sample of cancellous bone was fixed in a 2.5% glutaraldehyde solution at 4°C for 24 h, followed by decalcification with 10% ethylenediaminetetraacetic acid for 4 weeks. The decalcification solution was changed twice weekly to ensure complete removal of calcium from the sample surface. Subsequently, impregnation, embedding, and polymerization were performed using Epon812 resin for 12 h. Ultrathin sections measuring between 50 and 70 nm were double-stained with uranyl acetate and lead citrate before observation under a transmission electron microscope (JEM1220, Thermo Fisher Scientific).

### 2.6 Statistical analysis

Statistical analyses were performed using SPSS 23 software (IBM Corp., Armonk, NY, United States). Measurement data conforming to a normal distribution were presented as means ± standard deviations (x ± s), and T-tests were employed for analysis. For count data, either the chi-square test or Fisher’s exact test was used. The significance level was set at 0.05, with P < 0.05 considered statistically significant.

## 3 Results

### 3.1 Characterization of iodine-coated titanium needle

#### 3.1.1 Appearance and microscopic view of iodine-coated titanium needle

**FIGURE 1:**
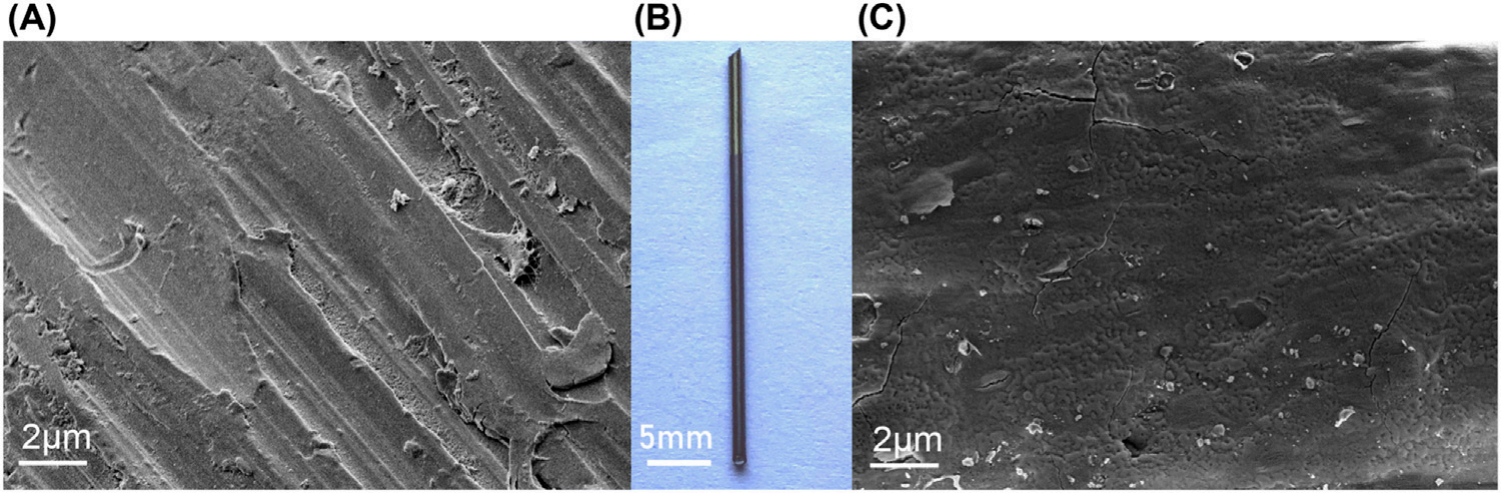
Appearance and microscopic analysis of the titanium needle. **(B)** Macroscopic view of titanium needle (3.0 cm in length, 2.0 mm in diameter, Ti-6Al-4V alloy): The uncoated titanium needle exhibits a golden-yellow surface, whereas the iodine-coated needle has a brown surface. Scanning electron microscope examination showing that **(A)** the uncoated titanium needle exhibits scattered scratches on its surface; **(C)** the iodine-coated titanium needle shows a dense coating with a uniform structure.

As shown in Figure 1, the uncoated titanium needle exhibited a golden-yellow surface, whereas the iodine-coated titanium needle exhibited a brown surface. The coating was uniformly distributed without any signs of surface collapse or thickening. Moreover, the coating demonstrated excellent stability as it remained intact even after ultrasonic cleaning. The scanning electron microscopy images revealed that the surface of the titanium needle was covered with a relatively dense and flat structure.

#### 3.1.2 XRF and EDS analysis

**FIGURE 2:**
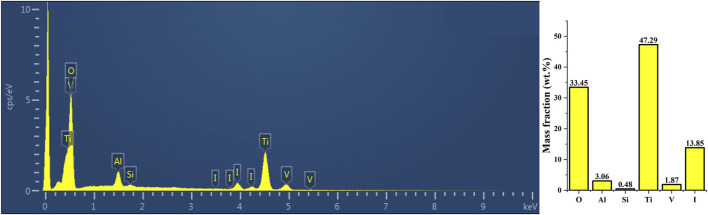
The XRF analysis results demonstrated the successful loading of iodine. The surface coating exhibited the highest elemental composition of titanium (47.29 wt%), oxygen (33.45 wt%), iodine (13.85 wt%), and aluminum (3.06 wt%). XRF, X-ray fluorescence spectroscopy.

**FIGURE 3:**
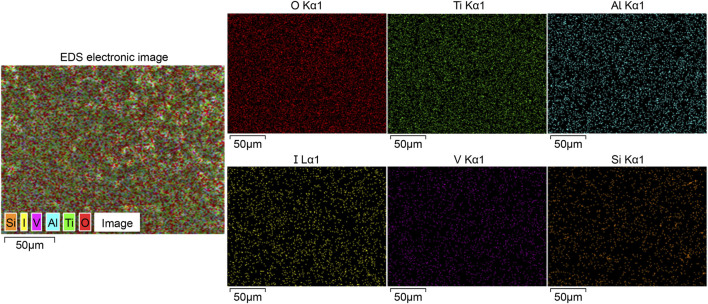
Outcomes of EDS analysis, revealing a uniform distribution of iodine and other elements across the surface of the iodine coating without any observed aggregation or shedding phenomena. EDS, energy dispersive spectrometry.

The XRF analysis results illustrating the successful loading of iodine are presented in Figure 2. The coating primarily comprised titanium (47.29 percentage by weight [wt%]), oxygen (33.45 wt%), iodine (13.85 wt%), and aluminum (3.06 wt%). Additionally, Figure 3 displays the results of EDS analysis, revealing a uniform distribution of iodine on the coating surface without any abnormal aggregation or shedding phenomena.

### 3.2 Antibacterial experiment *in vitro*

**FIGURE 4:**
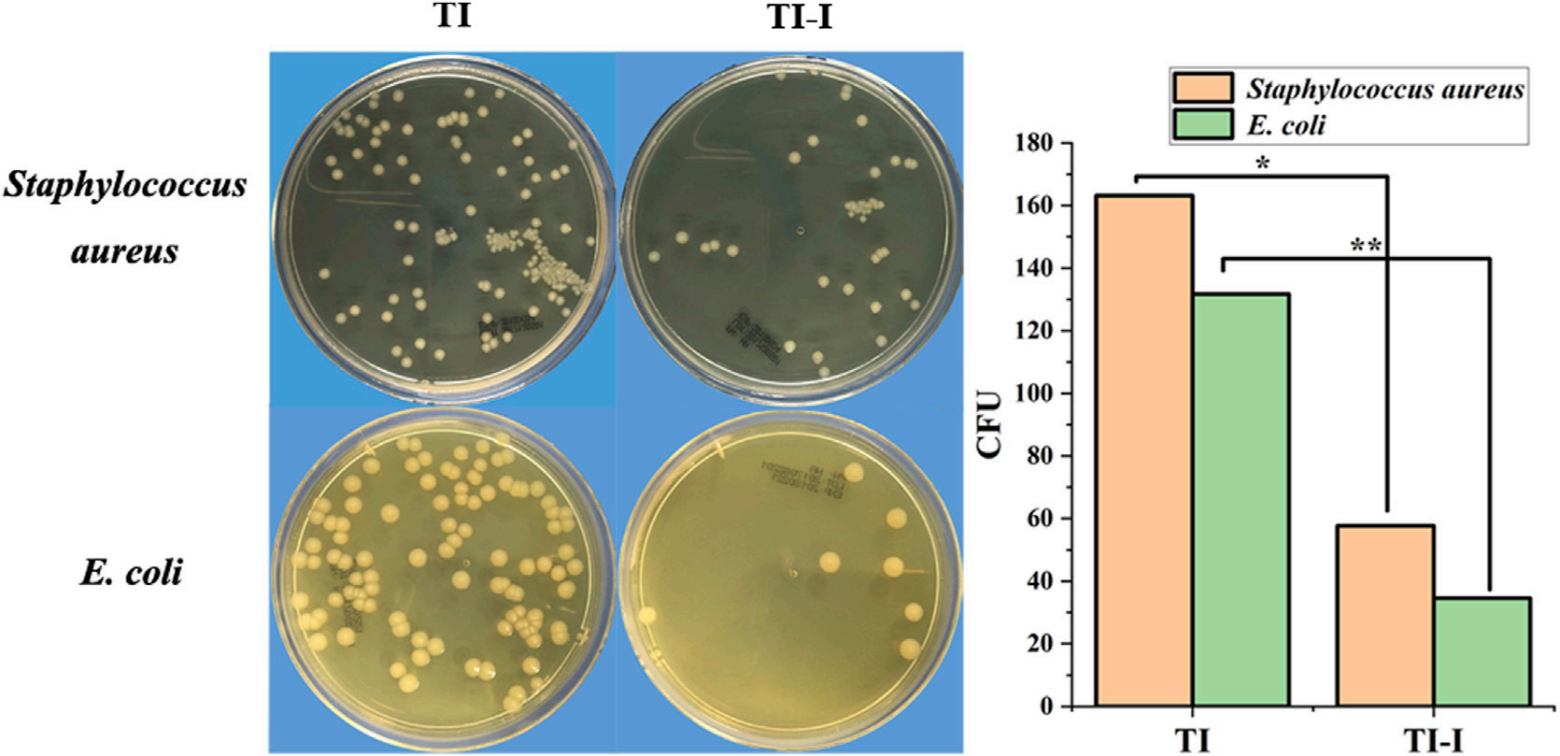
*In vitro* bacterial culture. The plate colony-forming unit (CFU) count of the *Staphylococcus aureus* standard strain in the control group (TI) and experimental group (TI-I) was 163.20 ± 21.54 and 57.70 ± 20.91, respectively, demonstrating statistical significance (*P < 0.05). For *Escherichia coli* standard strain, the plate CFU count was 131.70 ± 25.87 and 34.60 ± 19.60 in the control group (TI) and experimental group (TI-I), respectively, showing a significant difference (**P < 0.05). 11.67 wt.%.

The control (TI) and experimental groups (TI-I) were inoculated with the standard strain of *Staphylococcus aureus* (ATCC25923), coated, and cultured for 24 h. As shown in Figure 4, the CFU count on a single plate was 163.20 ± 21.54 and 57.70 ± 20.91, respectively, showing statistical significance (P = 0.000, <0.05). Similarly, after inoculation with the standard strain of *Escherichia coli* (ATCC25922), the CFU counts in the control (TI) and experimental group (TI-I) were found to be 131.70 ± 25.87 and 34.60 ± 19.60, respectively, following a 24-h period of plate coating and culture; this difference was statistically significant as well (P = 0.000, <0.05).

### 3.3 Release of iodine *in vitro*

**FIGURE 5:**
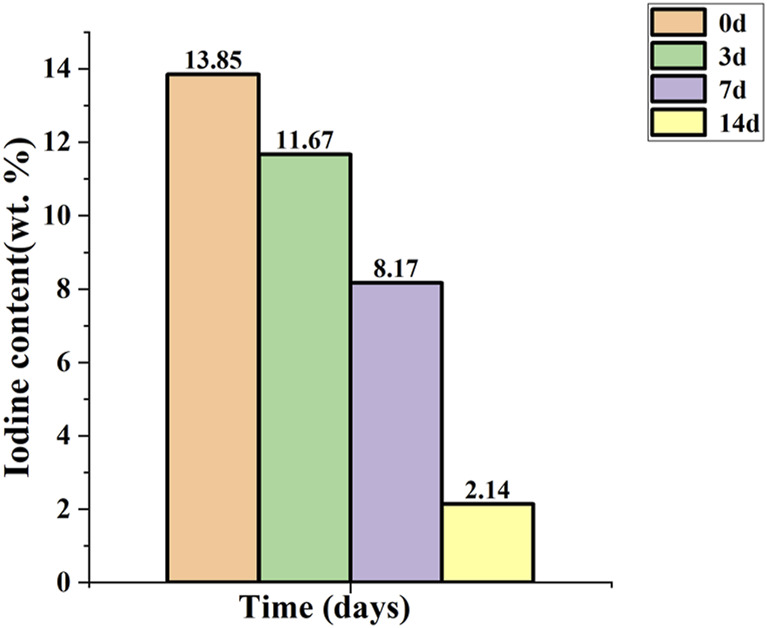
Release of iodine *in vitro*. The initial iodine content was 13.85 wt.%, which decreased to 11.67 wt.% on day 3, further declined to 8.17 wt.% on day 7, and subsequently decreased to 2.14 wt.% on day 14.

The titanium needle coated with iodine was stored in normal saline at a temperature of 37°C. Figure 5 illustrates the temporal variation in the iodine content. The measured iodine content was 11.67 wt.% on the 3rd day, decreased to 8.17 wt.% by the 7th day, and further declined to 2.14 wt.% by the 14th day.

### 3.4 Hemolysis test

**FIGURE 6:**
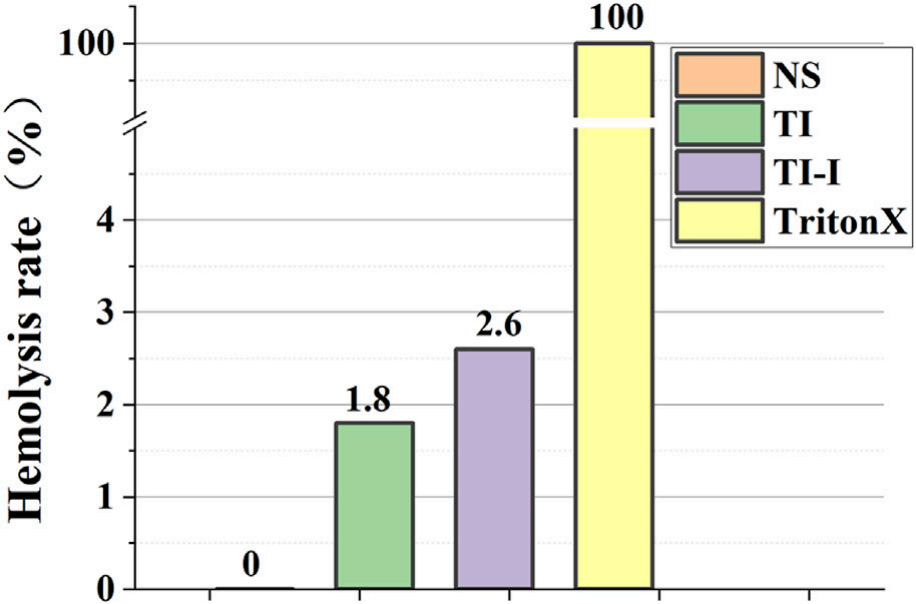
Hemolysis rate of red blood cells in model rabbits. The hemolysis rates of the ordinary titanium needles and iodine-coated titanium needles were 1.8% and 2.6%. Normal saline served as a negative control with a hemolysis rate of 0%, whereas the positive control using 1% TritonX-100 exhibited a hemolysis rate of 100%.

The hemolysis rates of the ordinary titanium needles and iodine-coated titanium needles were 1.8% and 2.6%, respectively, as depicted in Figure 6, both below the threshold of 5%. Normal saline served as a negative control with a hemolysis rate of 0%, whereas the positive control using 1% TritonX-100 exhibited a hemolysis rate of 100%.

### 3.5 *In vivo* experiments

#### 3.5.1 General situation assessment

All animals survived for 1 week after the experiment. In the control group, eight animals exhibited varying degrees of limited movement in the left lower extremity along with wound redness, swelling, exudation, and severe pus discharge. Conversely, all animals in the experimental group and the two control groups (Nos. 2 and 8) demonstrated normal lower-extremity activity and wound healing without any signs of redness, swelling, or suppuration. The wound conditions of some animals are shown in Supplementary Figure S2.

#### 3.5.2 Microbiological assessment

**FIGURE 7:**
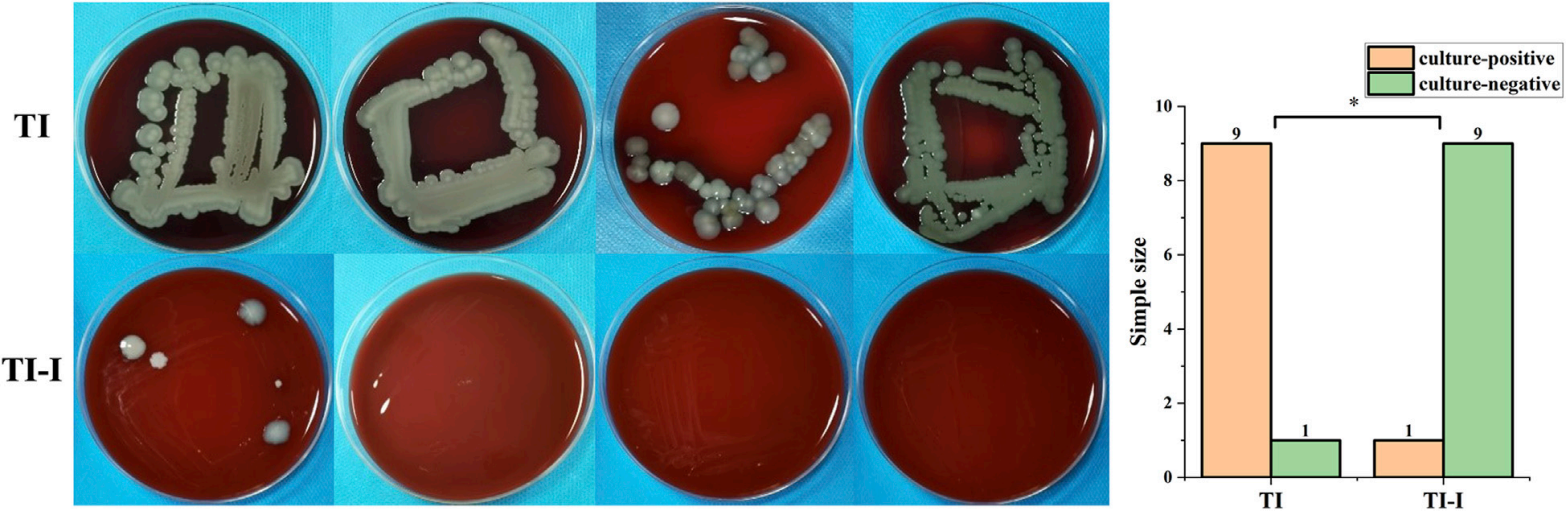
Microbial culture and statistical analysis. In the control group (TI), nine samples tested positive whereas one sample tested negative. Conversely, in the experimental group (TI-I), only one sample showed positive results with nine samples testing negative (*P < 0.05).

The culture results of secretions around the needle channels revealed that nine samples in the control group exhibited positive microbial cultures, whereas two samples showed negative results. In contrast, all nine samples from the experimental group displayed negative cultures, whereas six yielded positive outcomes. The statistical analysis and specific conditions of microbial culture are presented in Figure 7. Fisher’s exact test was employed to compare the results between the two groups at a significance level of α = 0.05, resulting in a p-value of 0.001, indicating statistical significance for P < 0.05.

#### 3.5.3 Histopathological evaluation of metaphyseal cancellous bone

**FIGURE 8:**
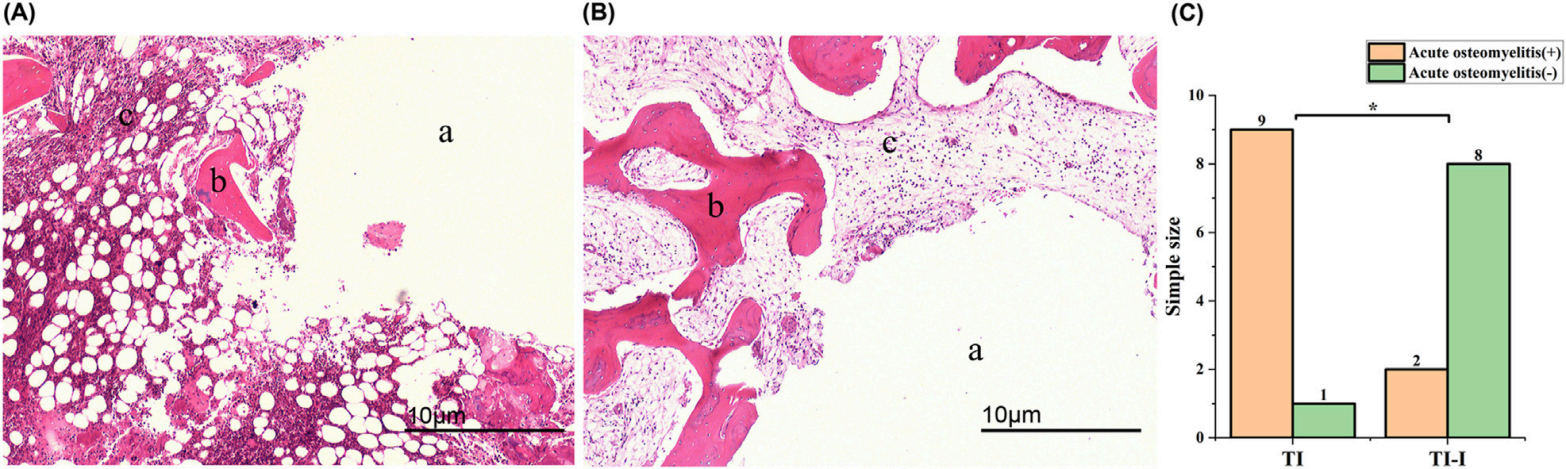
Histopathological features (magnification*40). **(A)** A prominent mass infiltration of neutrophils was observed, leading to local aggregation and formation of microabscesses, accompanied by bone destruction and fibrotic changes. **(B)** The bone tissue exhibited overall normal characteristics with no apparent inflammatory cell infiltration observed; however, mild fibrotic changes were noted. **(C)** Statistical analysis revealed a significant difference between the two groups (*P < 0.05). **(A)** needle track; **(B)** bone trabeculae and bone cells; **(C)** bone matrix. TI, control group, TI-I, experimental group.

Figure 8A shows nine samples from the control group and two samples from the experimental groups (experimental groups 2 and 6), showing various degrees of infection. Infection was characterized by significant aggregation of neutrophils within the bone marrow cavity, with more than 10 neutrophils clustered in each high-magnification field. Additionally, there was a limited presence of monocytes and pus cells forming microabscesses, accompanied by mild destruction and erosion of the bone tissue. Furthermore, cell-free fibrous necrotic fragments with eosinophilic properties were identified. The control group (No. 2 in the control group) and eight samples of the experimental group are depicted in Figure 8B, revealing mild fibrotic changes in the bone tissue without significant neutrophil aggregation. Jupiter scores and statistical analyses were conducted based on histopathological observations of the bone specimens. Quantitative analysis revealed a mean neutrophil count of 18.4 ± 3.2 cells/field in the control group (TI) versus 4.1 ± 1.8 cells/field in the experimental group (TI-I) (*P* < 0.001). The results demonstrated that scores greater than six points were observed in nine control groups and two experimental groups, accompanied by at least one of the four histopathological features indicative of acute osteomyelitis, thus meeting the diagnostic criteria for acute osteomyelitis. A comparison between the two groups is presented in Figure 8C, employing Fisher’s exact test with the significance level set at P = 0.005 (<0.05), indicating a statistically significant difference.

#### 3.5.4 Scanning electron microscopy observation

**FIGURE 9:**
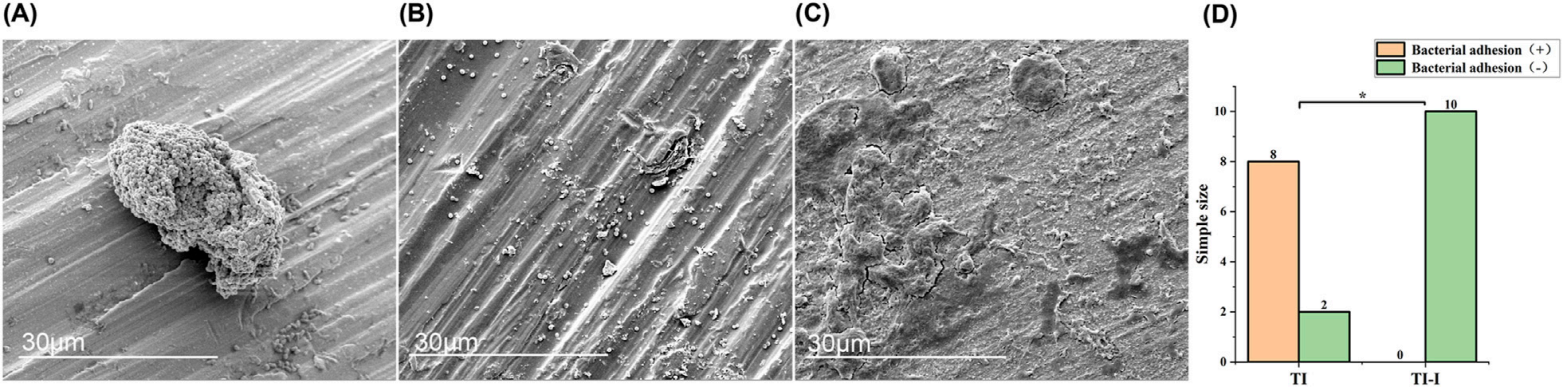
Morphology and statistical analysis of bacterial adhesion on implant surfaces. Scanning electron microscopy (magnification*5,000) revealed the following observations. **(A)** *Staphylococcus aureus* adhesion exhibited mostly irregular scattered and isolated distribution. **(B)** A significant number of *Staphylococcus aureus* formed clusters, displaying irregular shapes and local fusion into sheets. **(C)** No adhesion of *Staphylococcus aureus* was observed on the surface of the titanium needle. **(D)** Statistical analysis demonstrated a significant difference between the two groups, with *P < 0.05. TI, control group, TI-I, experimental group.

Scanning electron microscopy revealed that in the control group, *S aureus* adhesion was observed on the surface of eight ordinary titanium needles, with an irregularly scattered and isolated distribution (Figure 9A). Occasionally, local clusters of *Staphylococcus aureus* were observed, and a large number of *S. aureus* were clustered, irregularly shaped, and locally fused to sheets (Figure 9B). However, no *S aureus* was detected on the surface of the iodine-coated Kirchner needles in control groups 2, 5, and 10 (Figure 9C). The results for the two groups were compared, as shown in Figure 9D. A Fisher test was employed with a significance level set at 0.05 (P = 0.000), indicating a statistically significant difference at P < 0.05.

**FIGURE 10:**
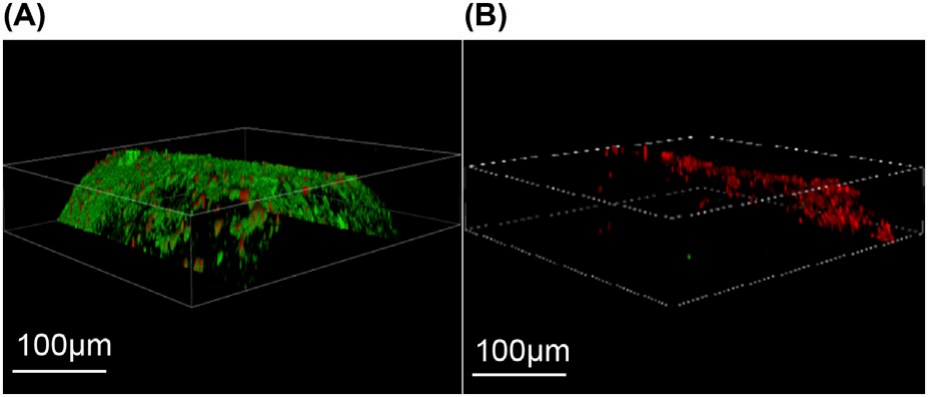
Laser confocal microscopy. **(A)** The control group exhibited a substantial number of densely distributed green fluorescence and sparsely scattered red fluorescence on the surface of Kirschner needles. **(B)** In the experimental group, iodine-coated titanium needles predominantly displayed a sparse distribution of red fluorescence with occasional small clusters of green fluorescence.

Confocal laser microscopy. Figure 10A shows the common Kirschner needle of the control group under confocal laser microscopy, revealing a dense distribution of live green fluorescent bacteria on its surface in the form of sheets or clumps. Additionally, a few locally scattered dead bacteria that exhibited red fluorescence were observed in a point-like pattern. In contrast, as depicted in Figure 10B, the iodine-coated Kirschner needles from the experimental group exhibited a sparser distribution of surface fluorescence, primarily consisting of dead bacteria with red fluorescence. A small number of live green fluorescent bacteria were sporadically dispersed within specific regions, displaying a punctate distribution.

**FIGURE 11:**
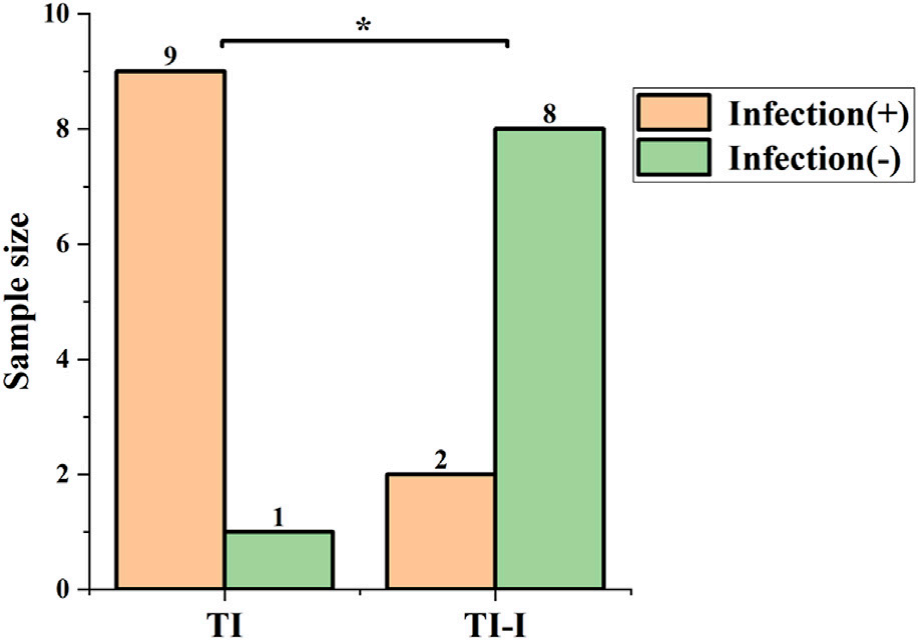
Infection rate. In the control group (TI), there were nine cases of infection, yielding an infection rate of 90%, while in the experimental group (TI-I), there were two cases of infection with an infection rate of 20%. The comparison between the two groups showed a statistically significant difference, *P < 0.05.

Infection rate evaluation. The statistical results of the infection rate evaluation are presented in Figure 11. All animals survived for 1 week post-surgery. In the control group (TI), there were nine cases of infection, resulting in an infection rate of 90%. Conversely, the experimental group (TI-I) had two cases of infection with an infection rate of 20%. Fisher’s exact test was used with the significance level set at 0.05. The obtained p-value was calculated as 0.005, indicating that P < 0.05 reached statistical significance.

#### 3.5.5 Morphological evaluation of metaphyseal bone cells with transmission electron microscopy

**FIGURE 12:**
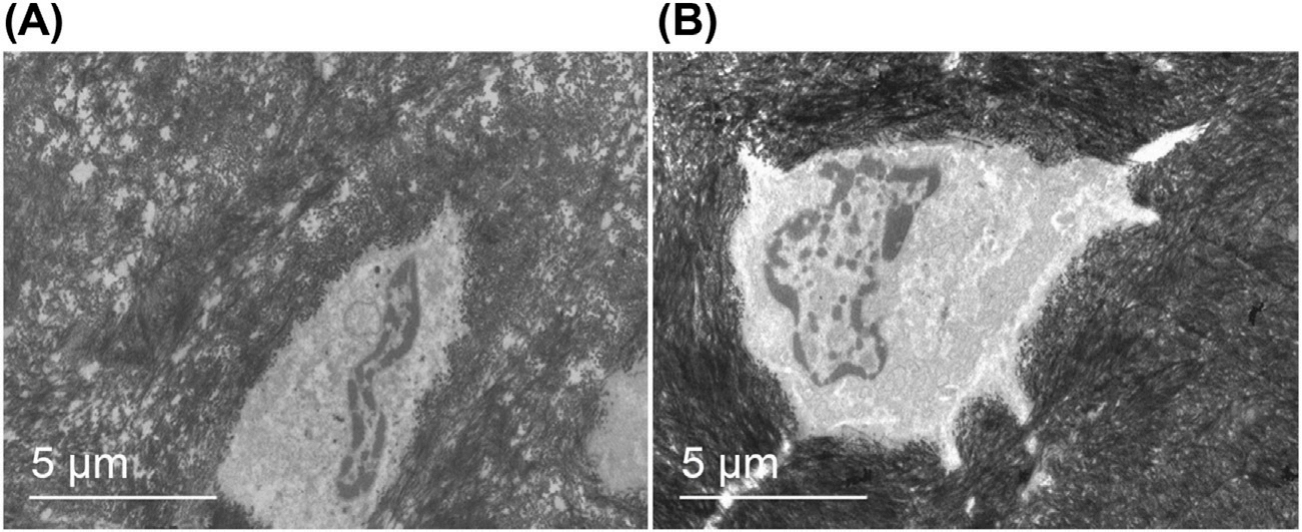
Morphology of metaphyseal bone cells. Transmission electron microscopy (magnification*5,000): **(A)** The bone matrix exhibited a high number of vacuoles with low density, aberrant morphology of bone cells, absence of bone tubules and cell processes, and reduced relative volume of the nucleus. **(B)** The bone matrix displayed uniform density, normal shape of bone cells, presence of bone tubules and cell processes, plump nuclei, and abundant contents.

The morphology of metaphyseal bone cells was examined using transmission electron microscopy, as depicted in Figure 12. In animals with acute bone infection (Figure 12A), a significant number of vacuoles were observed within the bone matrix, exhibiting a sparse density. In addition, abnormal bone cell morphology was evident, accompanied by the disappearance of both bone tubules and cell processes. Furthermore, a noticeable decrease in the relative volume of nuclei was observed. Conversely, in uninfected animals (Figure 12B), the density of the bone matrix appeared uniform and exhibited normal bone cell morphology. The bone tubules and cell processes are clearly visible, whereas the nuclei display fullness and richness in the material.

## 4 Discussion

Understanding the mechanisms underlying the development of bacterial biofilms represents a significant advancement in the investigation of orthopedic implant-associated infections in recent years. The challenging and recurrent nature of implant-associated infections can be attributed to biofilm formation, which occurs through a series of regulatory mechanisms following bacterial adhesion at the interface between the implant prosthesis and other internal implants (Foster et al., 2020). The formation of biofilms induces phenotypic changes in bacteria, thereby augmenting their resistance to multiple antibiotics and evading host immune defenses, leading to challenges in diagnosing biofilm-mediated periprosthetic infections and a lack of effective treatment options (Lamret et al., 2020; McConoughey et al., 2014). As such, the investigation of bacterial biofilms and the prevention and treatment of plant-associated infections in orthopedics have emerged as pressing tasks with significant clinical implications. Similar to other types of infections, prioritizing the prevention of biofilm-related periprosthetic infections is crucial. Current research efforts in this field predominantly focus on modifying the antibacterial coatings on internal implants to eradicate bacteria or impede their adhesion. Iodine, a halogen, has a long history of clinical antibacterial applications. It can destroy Gram-positive and Gram-negative bacteria, bacterial spores, fungi, protozoa, and some viruses (Lepelletier et al., 2020). These properties make iodine a good non-metallic antibacterial coating material.

Pioneering work by *Hiroyuki Tsuchiya*, *Toshiharu Shirai*, and their team since 2011 has greatly advanced the development of iodine-coated implants. Representative studies established a foundational technology involving iodine-doped titanium dioxide (TiO_2_) nanotube coatings (Shirai et al., 2011; Tsuchiya et al., 2012; Inoue et al., 2017; Inoue et al., 2019). This approach, developed at Chiba Institute of Technology, involves creating TiO_2_ nanotube structures on titanium surfaces followed by iodine doping within the nanotubes. While such coatings exhibit sustained antibacterial effects due to gradual iodine release, their inherent limitations include slow release kinetics and residual iodine compounds persisting on the implant surface for over a year (Yang et al., 2023). These retained compounds, trapped by the nanotube architecture, may interfere with long-term osseointegration and are suboptimal for acute infection prevention. To address this, our study introduces a pure iodine coating that bypasses TiO_2_ nanotube formation. By eliminating the nanotube structure, we achieved: rapid iodine release, higher iodine loading capacity, simplified fabrication. The rapid bactericidal effects of our iodine coating align with emerging strategies for acute infection prevention. Acute infections around orthopedic prostheses primarily manifest within 1–2 weeks post-implantation, coinciding with incision healing (Davidson et al., 2019). Our combined observations using these two techniques confirmed that iodine was evenly distributed throughout the coating. Although we considered the release cycle of iodine to be a concern, our *in vitro* release tests indicated that over 80% of the iodine is released from the coating surface within 2 weeks, creating an environment rich in iodine around the implant that promotes bacterial killing or inhibits bacterial adhesion on its surface. Acute infections around orthopedic prostheses primarily manifest before and after incision healing, necessitating heightened vigilance to prevent its occurrence during this critical period (Davidson, Spratt, and Liddle, 2019). The release of this iodine burst within a 2-week period would be beneficial in partially preventing acute infection around the prosthesis. Subsequently, as the incision healed, the incidence of infection further reduced. Compared to our previously studied iodine-doped titanium dioxide nanotube coating (Yang et al., 2023), the pure iodine coating exhibits enhanced iodine loading capacity and accelerated release kinetics, thereby offering greater advantages for acute periprosthetic infection management. Additionally, this approach circumvents any potential residual iodine compounds on the implant surface during later stages, thus minimizing their impact on bone integration.

The rapid-release iodine coating likely exerts its antibacterial effects through multiple synergistic mechanisms. First, iodine is a potent oxidizing agent that disrupts bacterial cell membranes and cytoplasmic structures by reacting with unsaturated fatty acids and thiol groups in proteins, leading to leakage of cellular contents and metabolic collapse (Barreto et al., 2020; Lepelletier et al., 2020). Second, the high local concentration of iodine released from the coating during the acute phase (peaking at 13.85 wt.% initially) creates a hostile microenvironment that inhibits bacterial adhesion and biofilm formation. This aligns with studies demonstrating iodine’s ability to interfere with bacterial quorum sensing and extracellular polymeric substance (EPS) production. Third, iodine’s broad-spectrum activity targets both Gram-positive (e.g., *Staphylococcus aureus*) and Gram-negative (e.g., *Escherichia coli*) bacteria, as evidenced by our *in vitro* assays, likely due to its penetration through cell walls and interaction with nucleic acids (Shirai et al., 2014).

Ensuring the safety of iodine-coated materials is a fundamental aspect of material research. Currently, toxicity assessment of drugs, chemicals, and materials primarily relies on *in vitro* hemolysis testing. The hemolysis rate observed for the iodine-coated materials during these tests was below 5%, thus meeting the biosafety standards set for such materials (Sæbø et al., 2023). In the field of orthopedic biomaterials, assessing the osteogenic properties and potential cytotoxicity towards bone cells can serve as crucial indicators to validate material safety. This research direction warrants further investigation for future advancements (Coppola et al., 2021). Subsequently, it is crucial to investigate whether the release of iodine induces alterations in trace elements within the body, consequently leading to changes in the internal environment and potential disease development. In particular, exploring if iodine influences thyroid hormone levels would be important. In a retrospective clinical study conducted by Shirai et al. (2023), the use of iodine-containing implants did not elicit any significant variations in hormone levels or impairments in thyroid function (Shirai et al., 2023). Further, in a prior animal experiment, iodine-containing biomaterials were implanted into rats and no discernible alterations in iodine or thyroid hormone levels were observed across various organs, tissues, and blood samples (Ueoka et al., 2021).

While this study demonstrates the efficacy of iodine coatings on small, standardized implants, scaling the technique to larger or anatomically complex prostheses (e.g., hip stems, porous acetabular cups) necessitates further investigation. Challenges include maintaining coating uniformity on curved or textured surfaces and adapting the electrophoretic deposition parameters (e.g., voltage gradients, electrolyte viscosity) to accommodate diverse geometries. Recent advances in EPD for orthopedic implants, such as multi-electrode setups and dynamic deposition protocols (Chouirfa et al., 2019), may provide pathways to address these challenges. Future work will focus on optimizing the process for clinical-grade implants and validating coating performance under biomechanical loading. The sterilization protocol is a critical consideration for iodine-coated implants. While low-temperature ethylene oxide fumigation preserved coating integrity in this study, standard hospital sterilization methods (e.g., steam autoclaving, gamma irradiation) may compromise iodine stability.

In the spectrum of bacteria causing infections around orthopedic prostheses, the majority of the identified bacteria exhibit drug resistance, with *Staphylococcus aureus* and *Escherichia coli* being the predominant pathogenic strains (Westberg et al., 2023; Kheir et al., 2018; Tai et al., 2022). As such, relevant standard strains of these two bacteria were selected for the *in vitro* antibacterial assays. However, the immune evasion capabilities of *Staphylococcus aureus* within the bone microenvironment and the protective mechanisms employed by biofilms are extremely intricate (Lamret et al., 2021). Therefore, this study investigated the antibacterial efficacy and safety of iodine-coated titanium needles in rabbits by creating a bone infection model around a metaphyseal prosthesis of *Staphylococcus aureus*. Analysis of the general conditions, bacterial culture, bone histopathology, scanning electron microscopy, and laser confocal microscopy results from both experimental groups revealed that the iodine-coated titanium needles had a lower infection rate than that with the ordinary titanium needles. This finding further confirmed the ability of the iodine coating to inhibit bacterial adhesion.

A combination of bone histopathological examination and microbial culture is widely regarded as the gold standard for diagnosing acute osteomyelitis. However, several factors, including preoperative antibiotic use, soft tissue contamination, sampling errors, and culture failures can limit the diagnostic value of microorganisms in identifying bone infections. Consequently, emphasizing the importance of histopathology is crucial in these cases (Sigmund et al., 2021). However, the histological criteria for acute osteomyelitis have not been clearly defined; as such, the diagnosis of acute osteomyelitis currently relies primarily on assessing neutrophil aggregate distribution, the presence of microabscesses in the bone marrow space, fibrinoid necrosis, and bacterial presence (Masters et al., 2019).

Using scanning electron microscopy, we observed the absence of *Staphylococcus aureus* adhesion to the surface of the iodine-coated titanium needles. However, in the imaging results obtained from laser confocal microscopy, we clearly visualized dead bacteria exhibiting red fluorescence and live bacteria exhibiting green fluorescence. Moreover, these live bacteria displayed a sparse distribution pattern, resembling spots. We conducted an analysis to explain this phenomenon: while local high-power scanning electron microscopy offers limited field of view with strong specificity but poor sensitivity; conversely, laser confocal three-dimensional imaging using staining provides a broader field of view with reduced specificity but enhanced sensitivity. Therefore, combining both detection methods in this study enabled a more direct visualization of the bacterial status on the coating surface.

In addition to biofilm formation on the surface of implants, the adhesion and colonization of bacteria in host tissues are crucial steps in the pathogenesis of implant-associated infections. Transmission electron microscopy was used to analyze a bone infection model caused by *Staphylococcus aureus*, which demonstrated the presence of bacteria within the infected bone tubules, bone lacunae, and bone cells. Due to the absence of defense mechanisms, bone cells serve as long-term reservoirs of *Staphylococcus aureus*. Prolonged inflammation and infection, along with bacterial virulence factors, induced alterations in bone cell morphology such as thinning of the bone matrix, loss of synapses, and nuclear shrinkage (Garcia-Moreno et al., 2022; Muthukrishnan et al., 2019). The acute periprosthesis bone infection model of *Staphylococcus aureus* was examined using transmission electron microscopy for approximately 1 week in the present study. Infection stimulation resulted in alterations in bone cell morphology in infected animals. Conversely, uninfected animals exhibited normal bone cells under transmission electron microscopy. The presence of plants within the iodine coating did not affect the bone cell morphology, indirectly confirming the biosafety of the iodine coating.

While this study did not explicitly measure surface energy or contact angle, prior research suggests that surface hydrophilicity and energy significantly influence bacterial adhesion. For instance, hydrophilic surfaces (low contact angle) are generally associated with reduced bacterial attachment due to weaker hydrophobic interactions between the surface and bacterial membranes (Tu et al., 2019). The iodine coating developed here, characterized by a dense and uniform structure (Figures 1–C), may exhibit altered surface hydrophilicity compared to uncoated titanium, potentially contributing to its observed antibacterial efficacy. Future studies will directly quantify these properties to elucidate their role in the coating’s performance.

Despite the strengths of this study, several limitations should be considered. Regarding toxicity, the safety of the material was only preliminarily assessed using an *in vitro* red blood cell hemolysis test. Future studies should investigate the effects of iodine-coated plants on osteocytes, osteoblasts, and bone marrow mesenchymal stem cells. Additionally, while this experiment demonstrated efficacy against acute bone infection models within 1 week, further experimental verification is necessary to determine whether prolonging the effect of iodine-coated plants will impact antibacterial results. This study did not assess surface energy or contact angle changes post-coating, which could further clarify the relationship between surface properties and antibacterial activity. Subsequent work will integrate these analyses to refine the coating design. Another limitation of this study is the reliance on qualitative descriptors (e.g., bacterial load via confocal/transmission/scanning electron microscopy) rather than quantitative metrics. The absence of standardized measurements, such as bacterial density per unit area or vacuole quantification, may reduce objectivity and statistical comparability. Future work will integrate image analysis software or algorithms to address this gap.

In this study, we coated the surfaces of orthopedic titanium implants via electrophoretic deposition, with confirmation of effective coating performed using scanning electron microscopy, XRF, and EDS X-ray spectroscopy. The safety of the material was preliminarily assessed using an *in vitro* hemolysis test. Subsequently, *in vitro* antibacterial experiments and a rabbit model of acute bone infection caused by *Staphylococcus aureus* around the prosthetic implants were conducted to demonstrate the superior antibacterial properties of the iodine-coated titanium implant. Overall, this study provides novel theoretical insights for the prevention and treatment of periprosthetic infections in orthopedics.

## Data Availability

The original contributions presented in the study are included in the article/Supplementary Material, further inquiries can be directed to the corresponding authors.

## Supplementary material

SUPPLEMENTARY FIGURE 1 Establishment of an experimental model for acute periprosthetic bone infection in the metaphyseal region of rabbit tibia. The procedural steps are as follows: Disinfection, Tibia drilling and grooving, insertion of titanium needle, inoculation with bacterial solution, closure of bone trough, and suturing.

SUPPLEMENTARY FIGURE 2 Wound appearance. The figure illustrates the local wound appearance of three rabbits in the control group (TI), exhibiting local redness, swelling, and purulent secretion exudation; whereas the subsequent figure displays the appearance of local wounds of three rabbits in the experimental group (TI-I), with no apparent signs of redness, swelling, or purulent exudate.
