# Supplementary figures and images for: A rapid-release pure iodine coating on titanium implants to mitigate acute periprosthetic infections
Source: Front Bioeng Biotechnol. 2025 Jul 10;13:1590411. doi: 10.3389/fbioe.2025.1590411 (PMC12287049; doi:10.3389/fbioe.2025.1590411)

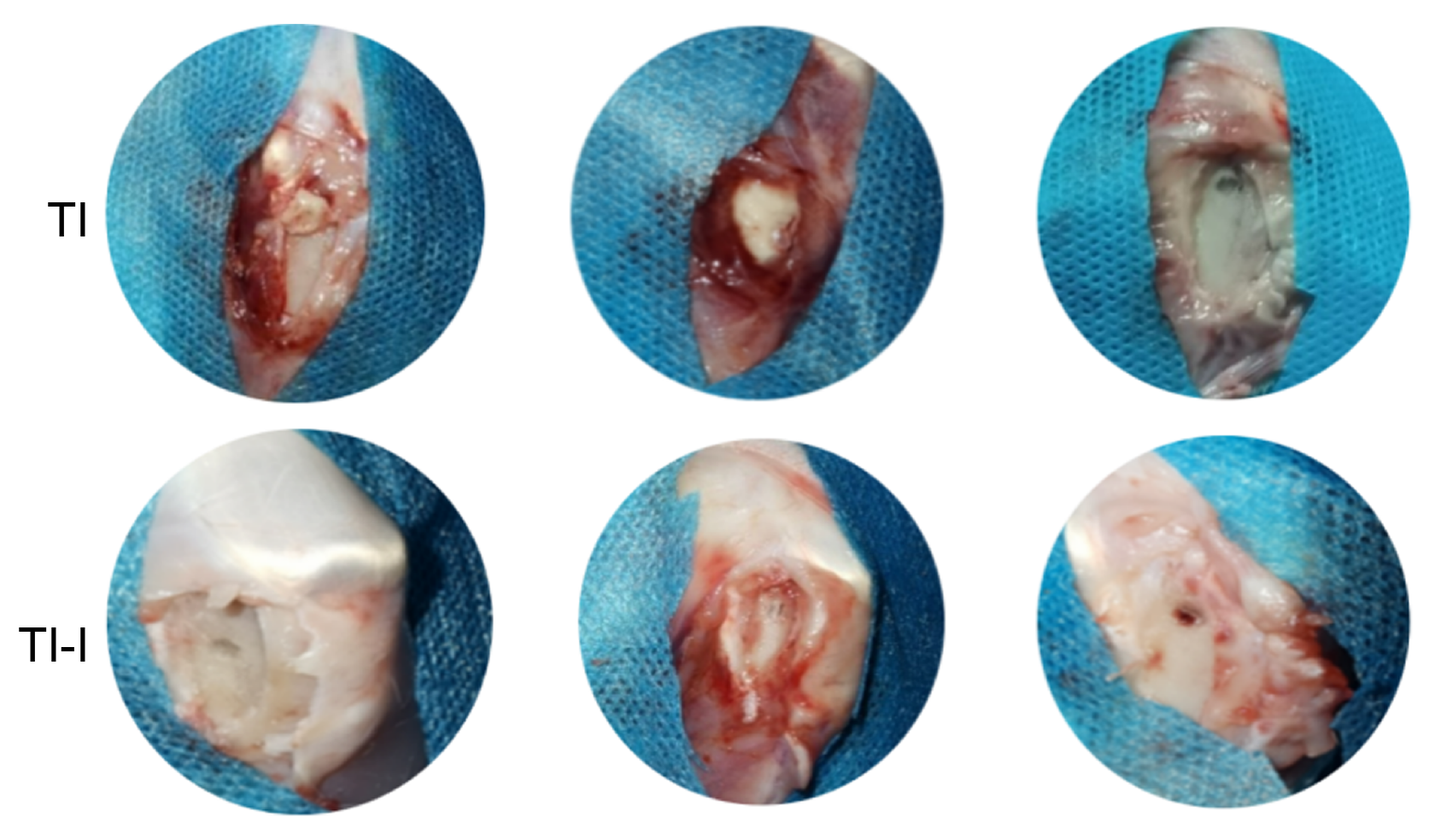

Supplement: Supplementary file 1 [file Image2.tif]

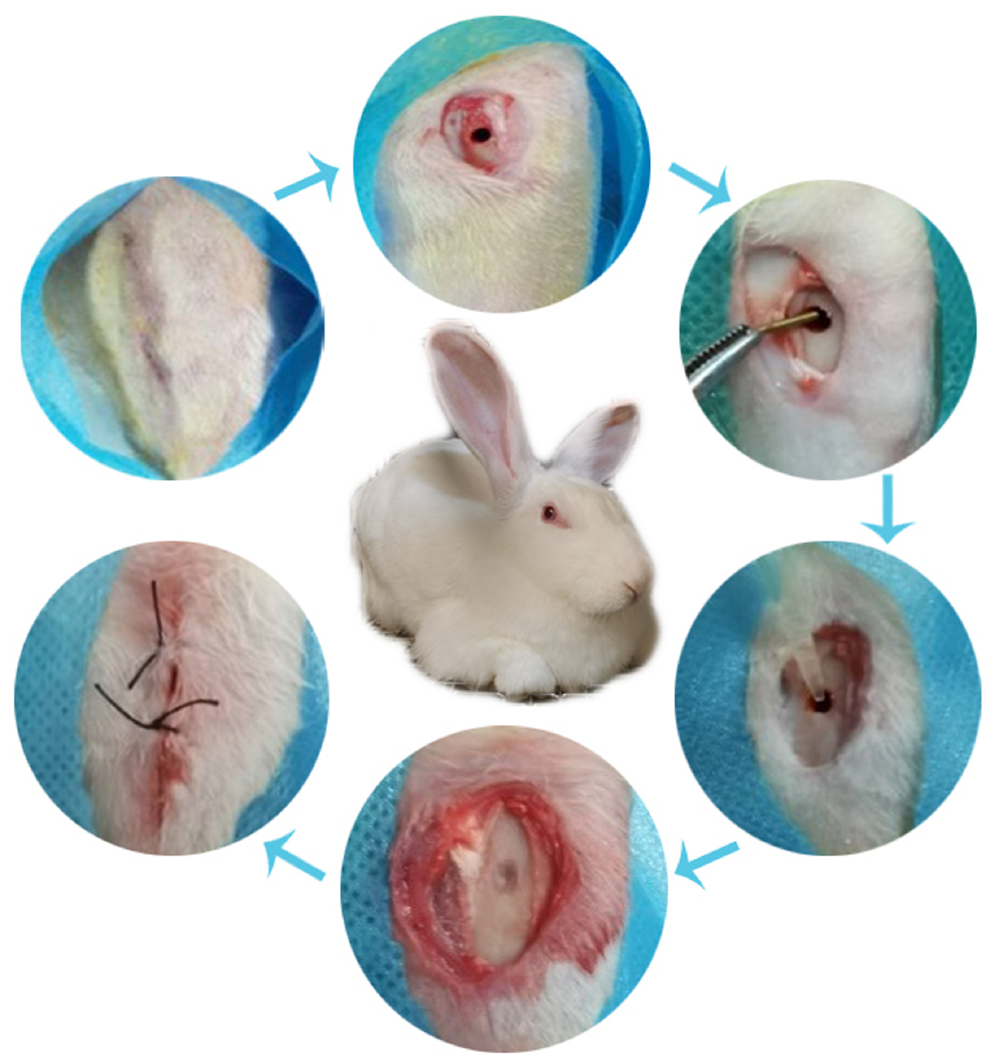

Supplement: Supplementary file 2 [file Image1.tif]
